# Free fibular strut graft in neglected femoral neck fractures in adult

**DOI:** 10.4103/0019-5413.45325

**Published:** 2009

**Authors:** Md Quamar Azam, AA Iraqi, MKA Sherwani, Amir Bin Sabir, M Abbas, Naiyer Asif

**Affiliations:** Department of Orthopaedic Surgery, J. N. Medical College, Aligarh Muslim University, Aligarh, Uttar Paradesh - 202 002, India

**Keywords:** Cancellous screw, femoral neck fracture, free fibular graft, neglected

## Abstract

**Background:**

Neglected femoral neck fracture in adults still poses a formidable challenge. Existing treatment options varies from osteotomy (with or without graft) to osteosynthesis using various implants and grafting techniques (muscle pedicle, vascularized, and nonvascularized fibula). The aim of this study was to assess outcome of nonvascularized fibular strut graft and cancellous screw fixation in neglected femoral neck fractures in the younger age group.

**Materials and Methods:**

Medical records of 32 patients of neglected femoral neck fracture, in the age group of 22-45 years (mean 37.8 years), operated between May 1994 to December 2001, were retrospectively reviewed. After the application of inclusion and exclusion criteria, 28 patients having three years minimum follow-up (mean 4.6 years) were included. Delay between injury and operation varied from four weeks to 42 weeks (mean 16.4 weeks). Closed reduction was achieved in 17 patients; open reduction through Watson-Jones anterolateral approach was performed in the remaining 15 patients in whom closed reduction failed. The fracture was transfixed with three parallel guide wires. Appropriate sized cannulated lag screw (7 mm) was then inserted in two of the wires. Selection of the third guide wire for fibula depended on the space available in both anteroposterior and lateral view.

**Results:**

Satisfactory bony union was obtained in 25 patients, of whom in four cases, the union occurred in 10-20° (mean 15°) of varus. Nonunion occurred in three patients (9.37%), and aseptic necrosis occurred in another six patients (18.75%). Of the 25 patients where union was achieved, five patients showed excellent results; 14 good and six had poor functional result, as evaluated using modified Anglen criteria.

**Conclusion:**

Nonvascularized fibular strut graft along with cancellous screws provides a dependable and technically less-demanding alternative procedure for neglected femoral neck fractures in young adults. Fibula being cortical provides mechanical strength besides stimulating the union and getting incorporated as biological graft.

## INTRODUCTION

Despite tremendous development in the implant design, imaging method, and surgical technique, the fractured neck of femur still poses a formidable challenge to the modern orthopedic surgeons. The incidence of complications like avascular necrosis of head of femur is reported to remain as high as 15-33%^1^,^2^ and that of nonunion is 10-30%. Fractured neck of femur, in younger population is reported to be 3-5% of the total femoral neck fractures, mostly as a result of high-velocity injuries like motor vehicle accident and fall from height. The greater force required for these fractures further increases the rate of complications^3^ to as high as 86%. Neglect of these injuries in developing countries^4^,^5^ along with other known factors such as precarious blood supply, inadequate reduction, and loss of fixation are the main reasons of nonunion and aseptic necrosis. Delay in surgery leads to variable degrees of neck absorption, proximal migration of distal fragment, and disuse osteoporosis. These factors together further make the task of achieving closed reduction and stable fixation difficult.^6^

There is no consensus regarding the treatment of neglected fractures of neck of femur in younger population. The management protocol, thus, remains a subject of controversy today as much as decades back. However, most agree that an attempt should be made to salvage the head. Existing treatment options include valgus osteotomy^1^,^7^ with or without bone graft and osteosynthesis^8^–^13^ using various implants and bone grafting techniques (muscle pedicle, free vascularized, or nonvascularized fibula). Valgus osteotomy does address the biomechanical factors; however, valgus orientation of the proximal femur decreases the abductor lever arm, resulting into persistent limp, and increases contact pressure on the head, which in turn leads to the rapid progress in avascular necrosis.^7^ Literature^4^,^6^ supports osteosynthesis using nonvascularized fibular strut graft in both fresh and old femoral neck fracture. We present here the outcome of nonvascularized fibular strut graft along with cancellous screw fixation in neglected femoral neck fractures.

## MATERIALS AND METHODS

Medical records of 32 patients of neglected femoral neck fracture, in the age group of 22-45 years (mean 37.8 years), operated between May 1994 to December 2001, were retrospectively reviewed. There were 22 men and 10 women, the male-to-female ratio being 2.2:1. The delay between the injury and operation varied widely from four weeks to 42 weeks (16.4 weeks). Twenty-eight of them with a minimum three-year follow-up (mean 4.6 years) were included [Table 1]. Patients having a minimum follow-up of less than three years, pathological fracture or showing features of osteonecrosis on plain radiograph were excluded from the study. An anteroposterior radiograph of hip was taken in 15° of internal rotation to visualize the whole profile of the neck. After admission, skeletal traction was applied to the proximal tibia. Radiographs of the hip were done every fifth day while on traction. In 17 patients, traction could pull down the distal fragment and maintain the neck shaft angle.

**Table 1:** Table of patients with case details

Case number	Age (years)/sex	Neglected period (weeks)	Shortening (preoperative) (cm)	Open/closed reduction	Follow-up (years)	Aseptic necrosis (postoperative)	Union (months)	Result
1	32/M	12	2.5	CR	7.6	Abs.	4.5	G
2	44/M	6	1.5	CR	6.4	Abs.	4	E
3	22/M	18	2.5	OR	7.5	Abs.	5	G
4	42/F	20	2	OR	5	Abs.	NU	NU
5	37/F	9	1.5	CR	5.5	Abs.	4	E
6	40/M	10	1.8	CR	4.2	Abs.	4.5	G
7	42/F	17	2.5	OR	6	Abs.	NU	NU
8	45/F	27	3	OR	4.5	Pres.	5	P
9	28/M	8	1.5	CR	Lost	Lost	Lost	Lost
10	25/M	36	2	OR	3	Pres.	5.5	P
11	25/F	14	1.5	CR	4.2	Abs.	4	E
12	40/M	42	2.5	OR	3.6	Pres.	7	P
13	32/M	6	1.5	CR	Lost	Lost	Lost	Lost
14	29/M	7	2	CR	3	Abs.	4.5	G
15	30/F	17	2.5	OR	4.5	Abs.	5	G
16	33/F	8	1.5	CR	4	Abs.	5	G
17	40/M	14	2	OR	5.2	Abs.	6	E
18	45/M	16	1.5	OR	4.2	Abs.	5	G
19	43/M	13	2.5	CR	7.6	Abs.	4.5	G
20	40/M	10	2.5	CR	3	Abs.	6.5	G
21	28/M	4	1.5	CR	Lost	Lost	Lost	Lost
22	26/M	30	2	OR	5.2	Pres.	6	P
23	34/F	7	1.5	CR	5	Abs.	5	G
24	42/F	24	2	OR	4	Abs.	NU	NU
25	41/M	12	2.5	CR	5	Abs.	5	G
26	23/M	6	1	CR	3.6	Abs.	4.8	E
27	39/M	5	1.5	CR	Lost	Lost	Lost	Lost
28	41/M	42	2.5	OR	3	Pres.	6.5	P
29	43/M	17	1.5	OR	4.6	Abs.	5	G
30	35/M	36	1.5	CR	3.2	Pres.	6	P
31	41/F	14	2.5	OR	3.5	Abs.	5.2	G
32	45/M	18	3	OR	3	Abs.	4.5	G

Pres. - Present; Abs. - Absent; CR - Close reduction; OR - Open reduction; Lat. - Anterolateral; NU - Nonunion; E - Excellent; G - Good; P - Poor

The operation was carried out on a standard operating table under image intensifer control. Closed reduction was achieved in 17 patients [Table 1] by gentle traction in 45° of flexion and in slight abduction; the hip was then extended and internally rotated to 30°-45°and brought parallel to the trunk. A Garden's index of 160° to 180° on the anteroposterior view and 0° to 20° on the lateral view were considered as acceptable for reduction and fixation. Since closed reduction failed, open reduction was performed through Watson-Jones anterolateral approach in 15 patients [Table 1]. Care was taken to incise the middle of the anterior capsule from the acetabular margin to 1 cm proximal of the intertrochanteric line to avoid major arterial circle around the base of the neck.

**Figure 1:**
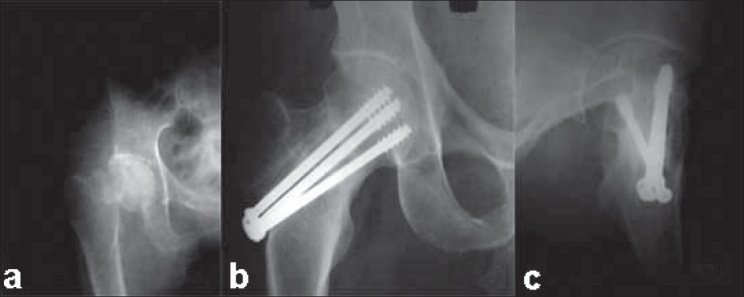
(a) Anteroposterior view of the right hip of a three months old femoral neck fractre, fixed with cancellous screw and nonvascularized fibular graft. Follow-up (anteroposterior (b) and lateral view (c)) radiograph at 18 months showing fracture union and graft incorporation.

**Figure 2:**
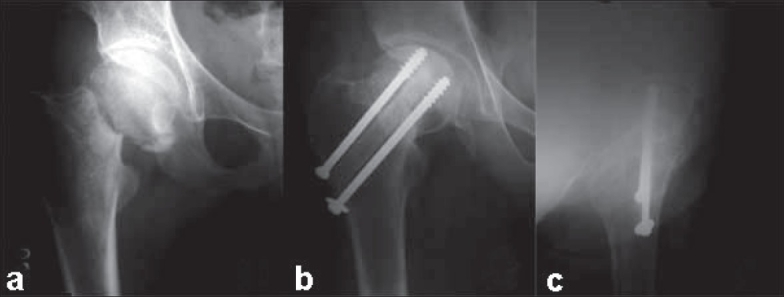
(a) Anteroposterior view of the right hip of a neglected femoral neck fracture (17 weeks old). Follow-up radiograph (anteroposterior (b) and lateral view (c)) showing union with partial incorporation of graft at the end of 30 months

After obtaining reduction, the fracture was transfixed with three parallel guide wires. Appropriate sized cannulated lag screw (7 mm) was then inserted in two of the wires. Selection of the third guide wire for fibula depended on the space available in both anteroposterior and lateral views. The required length of fibula was calculated by subtracting the protruded length of the guide wire from the total length (of the wire). One assistant then measured the length of the fibula, while the surgeon drilled over the third guide wire using Dynamic Condylar Screw (DCS) reamer for fibular placement. If required, the interosseus border of the fibula was nibbled to adjust the width to the femoral neck. In our first case, three cancellous screws and one fibular strut graft was used [Figure 1]; however, in the remaining cases [Figure 2], two cancellous screws and one fibular graft were used.

Non-weight-bearing crutch walking was started from fourth postoperative day; however, bed rest was advised for 3-5 weeks in cases where purchase of the screw was judged less than satisfactory because of either osteoporosis or various degrees of neck absorption. Patients were followed up every six weeks till union was achieved, then every three months until two years, and then six-monthly. Gradual full weight-bearing was permitted only when the radiographs (anteroposterior and lateral view) showed visible bony union.

The fracture was said to be united if the patient was walking without aid and the radiograph showed trabeculae bridging the fracture gap. Nonunion was defined as radiolucent gap existing between sclerosed bony ends even one year after the surgery. The functional result was evaluated using modified Anglen^14^ criteria. The outcome was graded as excellent, if there was no pain on full weight-bearing, no loss of motion greater than 20° in any plane, and less than 2-cm shortening. Occasional mild pain with activity, restriction greater than 20° in at least one plane, and shortening more than 2 cm was defined as a good outcome. Poor result meant that there was chronic pain requiring oral medication and ambulatory aid, with shortening more than 2 cm.

## RESULTS

Satisfactory bony union was achieved in 25 out of 28 patients. The time required for union varied from 4 to 7 months, average being 5.2 months. In four cases, union occurred in 10°-20° (mean 15°) of varus, because of gross osteoporosis and loss of reduction. Nonunion occurred in three cases and aseptic osteonecrosis in another six patients [Table 1]. Of the 25 patients where union was achieved, five showed excellent results, 14 patients showed good, and six had poor functional result as evaluated using modified Anglen's criteria [Table 1]. No major donor site morbidity was seen in any case; however, seven of our patients had minor complains such as mild ache, ankle swelling after rigorous walking, and some weakness of long toe flexors and extensors. This weakness resulted because of the loss of normal origin of long muscles; however, the donor site morbidity was not meticulously documented in each follow-up. Superficial infection was seen in three cases, but no deep infection was noted.

## DISCUSSION

Conservative treatment remained the method of choice until 1931, when Smith-Peterson *et al.*^15^ introduced the triflanged nail. King^16^ pioneered the use of fibular strut in combination with Smith-Peterson nail in cases of fractures of the neck of the femur. Several osteotomies have been described for old femoral neck fractures by Mc Murray's,^17^ Blount,^18^ Dickson,^19^ and Stewart *et al.*^20^ They concluded that realignment osteotomies gives most predictable result in young patients even in the presence of small areas of necrosis by modifying the mechanical environment about the fracture site, i.e., by converting the shearing forces into compressive forces. Dickson^19^ combined the cancellous bone grafting with an abduction osteotomy and reported encouraging results; however, he maintained that established femoral head necrosis was irreversible even after grafting. Marti *et al.*^21^ treated 41 patients with nonunion of femoral neck fracture and claimed healing in 86% cases. He further maintained that the improved biomechanics after osteotomy makes the bone grafting unnecessary. No cases of osteonecrosis were, however, noted postoperatively by Hartford *et al.*^9^ after intertrochanteric valgus osteotomy. Beris *et al.*^10^ further combined subtrochanteric valgus osteotomy and vascularized fibular graft in nonunion of the neck of the femur. Osteotomy, however, has important shortcomings such as persistent limp and increased rate of progress of osteoarthritis, attributable to the increase in contact pressure on the head of femur due to a decrease in the effective length of the abductor group of muscles. Furthermore, failure of osteotomy makes joint replacement significantly difficult.

Osteosynthesis using different techniques of bone grafting is described for neglected fractures of femoral neck. Our union rate of 89.28% is comparable to that of Nagi *et al.*^4^ (90%), Sandhu *et al.*^6^ (88.09%) and Le Croy *et al.*^3^ (90.90%), but inferior to that of Huang^2^ (100%) and Hou^11^ (100%). In the absence of an established classification system for neglected femoral neck fractures, the functional result is difficult to compare with the use of a different scoring system. However, 67.85% excellent to good result obtained in the present study is comparable to that of the previous studies.^2^,^4^

Hou^11^ noted that the iliac pedicle graft provides viable option, which hastens the fracture healing and also maintains head viability. Baksi^5^ and Meyers^13^ popularized muscle pedicle bone graft. Meyers *et al.*^13^ used quadratus femoris and sartorius muscle pedicle grafts or fibular graft in 136 patients, and the reported rate of nonunion and AVN was 11 and 3% respectively. Baksi^5^ treated 56 ununited intracapsular fractures by internal fixation and muscle pedicle grafting and obtained satisfactory union in 75% cases, delayed union in 7%, and nonunion in five cases. He concluded that muscle pedicle accelerates the healing of ununited fracture even in the presence of avascular head. Nagi *et al.*^4^ treated 16 old and 10 fresh fractures of neck of femur by open reduction and internal fixation with cancellous screw and nonvascularized fibular graft and reportedly achieved bony union in nine out of 10 nonunions. He also noted clinical and radiological improvement in four patients with preoperative avascular changes in the head. Nagi *et al.*^4^ stated that closed reduction was unlikely after three weeks, though we were able to obtain closed reduction in most of our cases up to eight weeks. This is attributable to preoperative skeletal traction for 1-2 weeks. Hip spica as recommended by Nagi *et al.*^4^ was not applied to any of our patients.

Previous literature^4^,^13^,^21^,^22^ stresses that osteonecrosis is not a contraindication of osteosynthesis. They observed that reaming provides internal autogenous graft and encourages growth of vascular granulation tissue. We agree with Sotto-Hall *et al.*^23^ that the femoral head is not necessarily osteonecrotic even with extended period of neglect, as such patients instinctively assume the position of maximum joint capacity (flexion, external rotation, and abduction), which relieves intraarticular tamponade.

Vascularized fibular graft^3^ and vascularized iliac bone graft^11^ are reported to give superior result; however, this consists of microvascular anastomosis that most orthopedic surgeons are not commonly well versed with. Leung and Shen^12^ obtained 100% union and satisfactory clinicoradiographic 5-7 years follow-up using vascularized iliac bone graft augmented by screw fixation. The use of nonvascularized fibular strut graft is technically less demanding. Fibula being cortical provides mechanical strength besides stimulating union, and its incorporation with the surrounding bone gives biological fixation. Once the graft is revascularized, the osteoblasts stimulated by bone morphogenic protein replace the resorbed bone. If this bone is appropriately stressed, the graft acquires sufficient strength to handle the observed forces. Minor morbidities such as mild ache and ankle swelling after rigorous walking were noted at the donor site, as found by Anderson *et al*^24^ and are attributed to the loss of origin of long muscles.

## CONCLUSION

Nonvascularized fibular strut graft along with cancellous screws provides a dependable and technically less-demanding alternative. We recommend its use to salvage the femoral head in younger patients. Fibula being cortical provides mechanical strength besides stimulating union and getting incorporated as biological graft.
